# 4-Meth­oxy-*N*-(2-nitro­benzyl­idene)aniline

**DOI:** 10.1107/S1600536808025117

**Published:** 2008-09-27

**Authors:** Xiao-Yan Ren, Fang-Fang Jian

**Affiliations:** a Microscale Science Institute , Weifang University, Weifang 261061, People’s Republic of China

## Abstract

The title compound, C_14_H_12_N_2_O_3_, was prepared by reaction of 2-nitro­benzaldehyde with 4-methoxy­benzenamine at 377 K. The molecule has an *E* configuration, with a dihedral angle between the two benzene rings of 43.3 (5)°. An intermolecular C—H⋯O interaction links molecules in zigzag chains down the *a* axis.

## Related literature

For the properties of Schiff bases, see: Deschamps *et al.* (2003 ▶); Tarafder *et al.* (2000 ▶); Rozwadowski *et al.* (1999 ▶). For related structures, see: Jian *et al.* (2006 ▶); Rozwadowski *et al.* (1999 ▶); Tarafder *et al.* (2000 ▶).
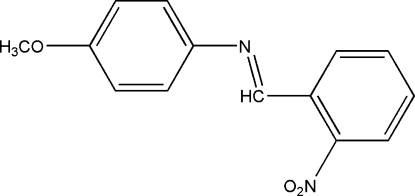


## Experimental

### 

#### Crystal data

C_14_H_12_N_2_O_3_
*M* *_r_* = 256.26
Orthorhombic, 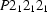

*a* = 4.0010 (8) Å
*b* = 7.8410 (16) Å
*c* = 40.447 (8) Å
*V* = 1268.9 (4) Å^3^
*Z* = 4
Mo *K*α radiation
μ = 0.10 mm^−1^
*T* = 293 (2) K
0.20 × 0.15 × 0.11 mm

#### Data collection

Bruker SMART CCD area-detector diffractometer
Absorption correction: none
3103 measured reflections
1651 independent reflections
748 reflections with *I* > 2σ(*I*)
*R*_int_ = 0.087

#### Refinement

*R*[*F* ^2^ > 2σ(*F* ^2^)] = 0.052
*wR*(*F* ^2^) = 0.135
*S* = 0.96
1651 reflections
173 parameters
H-atom parameters constrained
Δρ_max_ = 0.22 e Å^−3^
Δρ_min_ = −0.24 e Å^−3^

### 

Data collection: *SMART* (Bruker, 1997 ▶); cell refinement: *SAINT* (Bruker, 1997 ▶); data reduction: *SAINT*; program(s) used to solve structure: *SHELXS97* (Sheldrick, 2008 ▶); program(s) used to refine structure: *SHELXL97* (Sheldrick, 2008 ▶); molecular graphics: *SHELXTL* (Sheldrick, 2008 ▶); software used to prepare material for publication: *SHELXTL*.

## Supplementary Material

Crystal structure: contains datablocks global, I. DOI: 10.1107/S1600536808025117/at2599sup1.cif

Structure factors: contains datablocks I. DOI: 10.1107/S1600536808025117/at2599Isup2.hkl

Additional supplementary materials:  crystallographic information; 3D view; checkCIF report

## Figures and Tables

**Table 1:** Hydrogen-bond geometry (Å, °)

*D*—H⋯*A*	*D*—H	H⋯*A*	*D*⋯*A*	*D*—H⋯*A*
C14—H14*A*⋯O3^i^	0.93	2.63	3.469 (5)	146

Symmetry code: (i) 
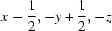
.

**Fig. 1.**
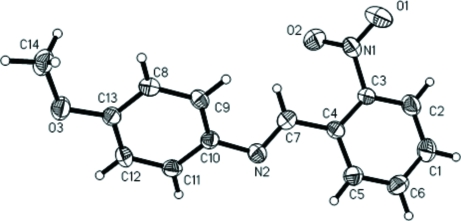
The molecular structure of the title compound with the atom-labeling scheme. Displacement ellipsoids are drawn at the 30% probability level.

## supplementary crystallographic information

### Comment

Schiff bases have antimicrobial (Tarafder *et al.*, 2000) and anticancer applications (Deschamps *et al.*, 2003). The recent growing interest in Schiff bases is also due to their ability to form intramolecular hydrogen bonds by electron coupling between acid-base centers (Rozwadowski *et al.*, 1999). The aim of our research is to find Schiff base with higher biological activity. Therefore we sythesized the title compound (I) and report its crystal structure here.

In the crystal structure of (I) (Fig. 1), the dihedral angle formed by the phenyl rings (C1–C6) and (C8–C13) was 43.3 (2)°. The C?N bond length [1.274 (5) Å] is in agreement with that observed before (Jian *et al.*, 2006). In the structure, there are no classical hydrogen bonds. Only, one intramolecular C–H···O type hydrogen bonding contact exists (Table 1).

### Experimental

A mixture of 2-nitrobenzaldehyde (0.02 mol) and 4-methoxybenzenamine (0.02 mol) was stirred with ethanol (50 mL) at 377 K for 5 h, affording the title compound (4.33 g, yield 84.5%). Single crystals suitable for X-ray measurements were obtained by recrystallization from acetone at room temperature.

### Refinement

H atoms were positioned geometrically and allowed to ride on their parent atoms, with C—H = 0.93 and 0.96 Å, and with *U*_iso_(H) = 1.2 or 1.5*U*_eq_ of the parent atoms. In the absence of significant anomalous scattering effects, Friedel pairs have been merged.

### Crystal data

**Table:**

C_14_H_12_N_2_O_3_	*F*(000) = 536
*M**_r_* = 256.26	*D*_x_ = 1.341 Mg m^−^^3^
Orthorhombic, *P*2_1_2_1_2_1_	Mo *K*α radiation, λ = 0.71073 Å
Hall symbol: P 2ac 2ab	Cell parameters from 1121 reflections
*a* = 4.0010 (8) Å	θ = 1.0–27.0°
*b* = 7.8410 (16) Å	µ = 0.10 mm^−^^1^
*c* = 40.447 (8) Å	*T* = 293 K
*V* = 1268.9 (4) Å^3^	Block, yellow
*Z* = 4	0.20 × 0.15 × 0.11 mm

### Data collection

**Table:**

Bruker SMART CCD area-detector diffractometer	748 reflections with *I* > 2σ(*I*)
Radiation source: fine-focus sealed tube	*R*_int_ = 0.087
graphite	θ_max_ = 27.0°, θ_min_ = 1.0°
phi and ω scans	*h* = −4→0
3103 measured reflections	*k* = −9→9
1651 independent reflections	*l* = 0→48

### Refinement

**Table:**

Refinement on *F*^2^	Secondary atom site location: difference Fourier map
Least-squares matrix: full	Hydrogen site location: inferred from neighbouring sites
*R*[*F*^2^ > 2σ(*F*^2^)] = 0.052	H-atom parameters constrained
*wR*(*F*^2^) = 0.135	*w* = 1/[σ^2^(*F*_o_^2^) + (0.0597*P*)^2^] where *P* = (*F*_o_^2^ + 2*F*_c_^2^)/3
*S* = 0.96	(Δ/σ)_max_ < 0.001
1651 reflections	Δρ_max_ = 0.22 e Å^−^^3^
173 parameters	Δρ_min_ = −0.24 e Å^−^^3^
0 restraints	Extinction correction: SHELXL, Fc^*^=kFc[1+0.001xFc^2^λ^3^/sin(2θ)]^-1/4^
Primary atom site location: structure-invariant direct methods	Extinction coefficient: 0.014 (3)

### Special details

**Table:**

Geometry. All e.s.d.'s (except the e.s.d. in the dihedral angle between two l.s. planes) are estimated using the full covariance matrix. The cell e.s.d.'s are taken into account individually in the estimation of e.s.d.'s in distances, angles and torsion angles; correlations between e.s.d.'s in cell parameters are only used when they are defined by crystal symmetry. An approximate (isotropic) treatment of cell e.s.d.'s is used for estimating e.s.d.'s involving l.s. planes.
Refinement. Refinement of *F*^2^ against ALL reflections. The weighted *R*-factor *wR* and goodness of fit *S* are based on *F*^2^, conventional *R*-factors *R* are based on *F*, with *F* set to zero for negative *F*^2^. The threshold expression of *F*^2^ > σ(*F*^2^) is used only for calculating *R*-factors(gt) *etc*. and is not relevant to the choice of reflections for refinement. *R*-factors based on *F*^2^ are statistically about twice as large as those based on *F*, and *R*- factors based on ALL data will be even larger.

### Fractional atomic coordinates and isotropic or equivalent isotropic displacement parameters (Å^2^)

**Table:**

	*x*	*y*	*z*	*U*_iso_*/*U*_eq_	
O3	0.5842 (9)	0.0957 (4)	0.02919 (6)	0.0602 (10)	
C10	0.8225 (12)	−0.3220 (5)	0.08753 (10)	0.0421 (12)	
N2	0.9205 (10)	−0.4713 (4)	0.10536 (8)	0.0506 (11)	
C13	0.6562 (12)	−0.0364 (5)	0.05006 (10)	0.0431 (11)	
C4	1.0942 (12)	−0.5998 (5)	0.15664 (10)	0.0433 (12)	
C11	0.8984 (13)	−0.3162 (5)	0.05408 (9)	0.0482 (12)	
H11A	1.0036	−0.4083	0.0440	0.058*	
C12	0.8172 (13)	−0.1730 (5)	0.03578 (10)	0.0505 (13)	
H12A	0.8723	−0.1689	0.0135	0.061*	
C3	1.2227 (13)	−0.5869 (5)	0.18867 (9)	0.0455 (12)	
C9	0.6573 (12)	−0.1861 (5)	0.10172 (10)	0.0438 (12)	
H9A	0.6033	−0.1899	0.1241	0.053*	
O2	1.4501 (11)	−0.3130 (4)	0.18665 (8)	0.0813 (13)	
C8	0.5695 (12)	−0.0423 (5)	0.08302 (10)	0.0471 (13)	
H8A	0.4543	0.0477	0.0927	0.056*	
N1	1.2977 (13)	−0.4173 (5)	0.20309 (10)	0.0623 (12)	
C6	1.1071 (14)	−0.9039 (5)	0.16521 (12)	0.0654 (16)	
H6A	1.0707	−1.0130	0.1569	0.078*	
C7	1.0088 (11)	−0.4530 (5)	0.13540 (10)	0.0460 (12)	
H7A	1.0199	−0.3436	0.1442	0.055*	
C5	1.0397 (13)	−0.7655 (5)	0.14548 (10)	0.0546 (14)	
H5A	0.9560	−0.7828	0.1243	0.065*	
C14	0.4282 (15)	0.2451 (5)	0.04323 (11)	0.0698 (16)	
H14A	0.3941	0.3287	0.0262	0.105*	
H14B	0.5701	0.2918	0.0601	0.105*	
H14C	0.2167	0.2143	0.0527	0.105*	
C2	1.2840 (15)	−0.7234 (6)	0.20893 (11)	0.0627 (16)	
H2B	1.3624	−0.7074	0.2303	0.075*	
O1	1.2071 (14)	−0.3908 (5)	0.23092 (9)	0.1181 (19)	
C1	1.2268 (16)	−0.8852 (6)	0.19681 (12)	0.0712 (17)	
H1A	1.2689	−0.9804	0.2099	0.085*	

### Atomic displacement parameters (Å^2^)

**Table:**

	*U*^11^	*U*^22^	*U*^33^	*U*^12^	*U*^13^	*U*^23^
O3	0.069 (2)	0.0526 (16)	0.0587 (19)	0.010 (2)	−0.0010 (19)	0.0113 (16)
C10	0.040 (3)	0.040 (2)	0.047 (3)	−0.006 (3)	−0.006 (3)	0.001 (2)
N2	0.054 (3)	0.052 (2)	0.046 (2)	−0.002 (2)	−0.007 (2)	0.0026 (18)
C13	0.043 (3)	0.042 (2)	0.045 (3)	−0.002 (3)	−0.007 (3)	0.000 (2)
C4	0.043 (3)	0.048 (2)	0.039 (2)	0.002 (3)	0.003 (2)	−0.003 (2)
C11	0.054 (3)	0.046 (2)	0.044 (3)	−0.004 (3)	0.006 (3)	−0.009 (2)
C12	0.056 (4)	0.058 (3)	0.037 (2)	−0.001 (3)	0.001 (3)	0.007 (2)
C3	0.053 (3)	0.044 (2)	0.040 (2)	−0.003 (3)	0.001 (2)	−0.004 (2)
C9	0.041 (3)	0.053 (2)	0.038 (2)	0.003 (3)	−0.007 (2)	0.001 (2)
O2	0.103 (4)	0.064 (2)	0.078 (2)	−0.020 (3)	−0.007 (3)	−0.0003 (19)
C8	0.048 (3)	0.048 (3)	0.045 (3)	−0.001 (3)	−0.001 (3)	−0.006 (2)
N1	0.077 (3)	0.067 (3)	0.043 (2)	0.003 (3)	−0.003 (3)	0.002 (2)
C6	0.079 (4)	0.045 (3)	0.072 (3)	0.000 (3)	−0.007 (3)	−0.001 (3)
C7	0.037 (3)	0.045 (3)	0.056 (3)	−0.001 (2)	0.000 (3)	0.000 (2)
C5	0.060 (4)	0.050 (3)	0.054 (3)	0.001 (3)	−0.007 (3)	0.000 (2)
C14	0.073 (4)	0.055 (3)	0.081 (3)	0.010 (4)	−0.019 (3)	0.014 (3)
C2	0.078 (4)	0.064 (3)	0.047 (3)	0.014 (3)	−0.005 (3)	0.005 (2)
O1	0.181 (5)	0.108 (3)	0.065 (2)	−0.024 (4)	0.010 (3)	−0.029 (2)
C1	0.089 (4)	0.061 (3)	0.064 (3)	0.012 (4)	−0.010 (4)	0.017 (3)

### Geometric parameters (Å, °)

**Table:**

O3—C13	1.367 (4)	C9—C8	1.402 (5)
O3—C14	1.444 (5)	C9—H9A	0.9300
C10—C9	1.379 (5)	O2—N1	1.217 (5)
C10—C11	1.387 (5)	C8—H8A	0.9300
C10—N2	1.430 (5)	N1—O1	1.201 (5)
N2—C7	1.274 (5)	C6—C1	1.373 (6)
C13—C12	1.376 (5)	C6—C5	1.373 (5)
C13—C8	1.379 (5)	C6—H6A	0.9300
C4—C5	1.393 (5)	C7—H7A	0.9300
C4—C3	1.398 (5)	C5—H5A	0.9300
C4—C7	1.477 (5)	C14—H14A	0.9600
C11—C12	1.384 (5)	C14—H14B	0.9600
C11—H11A	0.9300	C14—H14C	0.9600
C12—H12A	0.9300	C2—C1	1.378 (6)
C3—C2	1.370 (5)	C2—H2B	0.9300
C3—N1	1.482 (5)	C1—H1A	0.9300
			
C13—O3—C14	117.6 (3)	C9—C8—H8A	120.5
C9—C10—C11	119.0 (4)	O1—N1—O2	123.2 (5)
C9—C10—N2	123.7 (4)	O1—N1—C3	117.5 (4)
C11—C10—N2	117.3 (4)	O2—N1—C3	119.3 (4)
C7—N2—C10	117.7 (3)	C1—C6—C5	121.7 (4)
O3—C13—C12	115.4 (4)	C1—C6—H6A	119.2
O3—C13—C8	124.7 (4)	C5—C6—H6A	119.2
C12—C13—C8	119.8 (4)	N2—C7—C4	122.1 (4)
C5—C4—C3	115.2 (4)	N2—C7—H7A	119.0
C5—C4—C7	120.2 (4)	C4—C7—H7A	119.0
C3—C4—C7	124.6 (4)	C6—C5—C4	121.2 (4)
C12—C11—C10	119.8 (4)	C6—C5—H5A	119.4
C12—C11—H11A	120.1	C4—C5—H5A	119.4
C10—C11—H11A	120.1	O3—C14—H14A	109.5
C13—C12—C11	121.1 (4)	O3—C14—H14B	109.5
C13—C12—H12A	119.4	H14A—C14—H14B	109.5
C11—C12—H12A	119.4	O3—C14—H14C	109.5
C2—C3—C4	124.3 (4)	H14A—C14—H14C	109.5
C2—C3—N1	115.4 (4)	H14B—C14—H14C	109.5
C4—C3—N1	120.3 (4)	C3—C2—C1	118.5 (4)
C10—C9—C8	121.1 (4)	C3—C2—H2B	120.8
C10—C9—H9A	119.4	C1—C2—H2B	120.8
C8—C9—H9A	119.4	C6—C1—C2	119.2 (5)
C13—C8—C9	119.1 (4)	C6—C1—H1A	120.4
C13—C8—H8A	120.5	C2—C1—H1A	120.4

### Hydrogen-bond geometry (Å, °)

**Table:**

*D*—H···*A*	*D*—H	H···*A*	*D*···*A*	*D*—H···*A*
C14—H14A···O3^i^	0.96	2.63	3.469 (5)	146.

Symmetry codes: (i) *x*−1/2, −*y*+1/2, −*z*.
